# Predictive Simulation of Process Windows for Powder Bed Fusion Additive Manufacturing: Influence of the Powder Bulk Density

**DOI:** 10.3390/ma10101117

**Published:** 2017-09-22

**Authors:** Alexander M. Rausch, Vera E. Küng, Christoph Pobel, Matthias Markl, Carolin Körner

**Affiliations:** 1 Chair of Materials Science and Engineering for Metals (WTM), Department of Materials Science and Engineering, Friedrich-Alexander-Universität Erlangen-Nürnberg (FAU), Martensstr. 5, D-91058 Erlangen, Germany; vera.kueng@fau.de (V.E.K.); matthias.markl@fau.de (M.M.); carolin.koerner@fau.de (C.K.); 2 Joint Institute of Advanced Materials and Processes (ZMP), Friedrich-Alexander-Universität Erlangen-Nürnberg (FAU), Dr.-Mack-Str. 81, D-90762 Fürth, Germany; christoph.pobel@fau.de

**Keywords:** additive manufacturing, selective electron beam melting, numerical simulation, Ti-6Al-4V, porosity, powder density, Lattice Boltzmann Method, defect statistics, process map

## Abstract

The resulting properties of parts fabricated by powder bed fusion additive manufacturing processes are determined by their porosity, local composition, and microstructure. The objective of this work is to examine the influence of the stochastic powder bed on the process window for dense parts by means of numerical simulation. The investigations demonstrate the unique capability of simulating macroscopic domains in the range of millimeters with a mesoscopic approach, which resolves the powder bed and the hydrodynamics of the melt pool. A simulated process window reveals the influence of the stochastic powder layer. The numerical results are verified with an experimental process window for selective electron beam-melted Ti-6Al-4V. Furthermore, the influence of the powder bulk density is investigated numerically. The simulations predict an increase in porosity and surface roughness for samples produced with lower powder bulk densities. Due to its higher probability for unfavorable powder arrangements, the process stability is also decreased. This shrinks the actual parameter range in a process window for producing dense parts.

## 1. Introduction

Over the last decades, the interest in additive manufacturing technologies has grown in industry as well as in the scientific community [1], as these technologies allow the manufacturing of near net-shape and complex geometries. The most common powder bed fusion technologies are selective electron beam melting (EBM) and selective laser melting (SLM) [2]. To build parts with sufficient mechanical properties, reliable process parameters are necessary. However, powder bed statistics and melt pool dynamics are major uncertainties that deeply influence process stability [3]. Therefore, it is necessary to find reliable process parameters and understand their influence on the final density.

Process windows identify a process parameter range for sound materials. Many different parameters, such as beam power, beam width, scan velocity, line offset, layer thickness, etc. determine the final result. For process windows, the beam power and scan velocity are often varied while keeping all other parameters constant. The quantity of interest can be the relative density of produced parts [4,5,6,7,8,9,10], the shape of single melt tracks [11,12,13,14,15,16], the microstructure [17], or qualitative criteria such as balling and delamination effects [18] or surface quality and non-quantitative porosity analysis [19]. In any case, the goal is to improve process stability and to provide final parts with sufficient mechanical properties. Numerical studies are an effective support for finding a suitable process window [18,20,21]. Because the underlying physical mechanisms are hardly observable, in many cases simulations are the only way to identify and understand the direct influence of melt pool dynamics, evaporation effects, or the powder bed during the manufacturing process [22].

The stochastic uncertainties in the deposited powder layer depend on the powder size distribution, its bulk density, and particle shape. They have a non-negligible influence on the relative density of the final part. Zielinski et al. [23] numerically examined the influence of regular and irregular powder arrangements on the porosity. They identified the statistical unpredictability in powder layer formation as a key factor for producing dense parts. Gürtler et al. [24] conducted numerical simulations and found an influence of defects in the powder layer on the final relative density. Additionally, Spierings et al. [25,26] showed the impact of the powder size distribution on the relative density by comparing different powders for SLM. Similarly, Liu et al. [27] identified the importance of the powder layer density for minimizing porosity. Furthermore, Körner et al. [15] found that a defined melt pool shape can be achieved by increasing the powder bulk density of a stochastic powder bed.

Based on all these studies, it is clear that a macroscopic numerical approach where the powder bed is described by a continuum approach cannot represent the influence of a stochastic powder bed. Some mesoscopic numerical setups deal with the melting of a completely resolved powder bed [21,24,28,29,30,31,32,33]. However, none of them study the successive building of many layers. This is necessary to identify the statistics of powder bed-related defects and their propagation, as it can be seen for channel-like porosities [34].

The purpose of this work is to use numerical simulations for the investigation of defect structures identified by experiments and to examine the influence of the powder bulk density on these defects and the resulting process window. The numerical method includes full melt pool dynamics. Furthermore, the randomly packed powder bed is described explicitly. To capture the statistics of defects, 100 layers, each with 101 scan lines, are built in the simulations, which represents a build height that was not achieved in mesoscopic simulations until now. As an example, a selective electron beam melted Ti-6Al-4V process window is chosen. In a first step, the simulation tool is validated by comparing the simulated and experimental process windows. The focus lies on the transition zone from porous to dense parts. Finally, the influence of the powder bulk density on defect formation and the process window is studied numerically.

## 2. Materials and Methods

### 2.1. Experimental Setup

The influence of process parameters on part densities is investigated for cuboid samples built on an Arcam Q10 EBM machine (Arcam AB, Mölndal, Sweden). Grade 23 argon-gas-atomized Ti-6Al-4V ELI powder (TLS Technik GmbH & Co. Spezialpulver, Bitterfeld-Wolfen, Germany) was used with particle diameters between 45 and 105 μm. The powder has a logarithmic Gaussian distribution with a mean diameter $d_{50}$ of 70.8 μm. The apparent density of the powder was measured with the funnel method [35]. Based on the apparent density, a powder bulk density of 55% was calculated. The powder was applied on the build platform by a rake with a nominal layer thickness $l_{\mathrm{nom}}$ of 50 μm and preheated with a defocused electron beam. The preheating temperature measured at the bottom of the build platform was 650 ${}^{\circ }C$. The whole process was performed under vacuum conditions with an additional helium partial pressure of 7 × 10^−3^ mbar.

After preheating the whole powder bed, the cuboid’s ground area of 15 × 15 mm${}^{2}$ was melted with a snake-like hatching strategy by the focused electron beam with a line offset of 50 μm. Afterwards, the build platform was lowered by about the nominal layer thickness and a new powder layer was applied. For each successive layer, the hatching direction was rotated by 90${}^{\circ }$. The final sample height was 10 mm or 15 mm, and all samples were built on supports.

The scan velocity *v* and the beam power *P* were varied from 0.4 to 40 m/s and 30 to 3000 W, respectively. The acceleration voltage was constant at 60 kV and only the electric current was altered. The process parameter range is based on a corresponding process window determined by Scharowsky et al. [7] with a different EBM machine (Arcam S12, Arcam AB, Mölndal, Sweden). Neither here, nor in [7] a design of experiments was conducted. The definition of the line energy $E_{l}=P/v$ instead of the beam power *P* is used to present the results.

One sample was prepared for each parameter combination. The exact combinations can be seen in Figure 4. In total, 160 samples were built. Each sample was cut in half along the built direction. One half of each sample was ground and chemomechanically polished. These microsections were used to obtain the relative density via conventional optical microscopy. The density was determined 2 mm beneath the top surface of the samples over the whole width perpendicular to the built direction. Further variance analysis was not performed. The waviness of the top surface was evaluated by optical inspection. A more detailed description of the principal EBM process and the sample preparation methods can be found in [5,7].

### 2.2. Numerical Setup

The numerical framework is a 2D mesoscopic approach based on the Lattice Boltzmann method (LBM) designed for the simulation of powder bed fusion additive manufacturing processes [15]. It is capable of describing powder particle deposition [15], beam absorption [36], melt pool dynamics including wetting and capillarity [37], evaporation [38] with phase composition changes [39] and grain growth [40]. The LBM is a computational fluid dynamics method that does not solve the Navier–Stokes equation directly, but follows the microscopic Boltzmann equation with distribution functions. Apart from the discretization of space and time, the fluid velocity is also discretized in nine directions (2D), where each has a corresponding distribution function. The macroscopic fluid properties can be calculated as moments of these distribution functions. Due to this discretization, the LBM is highly local and is suited for free and complex fluid surfaces. In this model we use a two distribution function method: The hydrodynamic movement is calculated via a 2D LBM framework with a volume of fluid approach for free surfaces [41]. The passive scalar model of Zhang et al. [42] is used to determine the thermal field. For a closer description of the basic model see [15].

**Figure 1:**
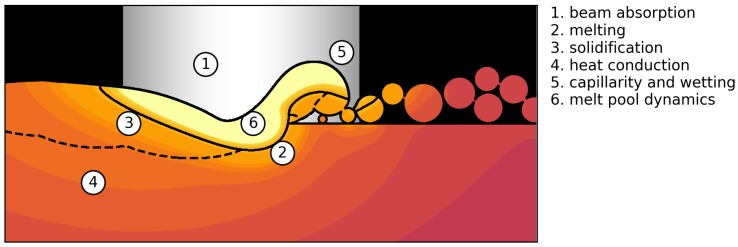
Physical phenomena involved in a powder bed fusion additive manufacturing process. The figure depicts an example simulation plane with beam movement out of the plane. Melt pool and already solidified material are indicated. The colored temperature field is shown qualitatively.

Since the focus of this paper is the transition zone of a process window from porous to dense parts, evaporation effects and phase composition changes were neglected as they are only relevant for higher beam powers at the upper limit of process window. Figure 1 illustrates the setup by an example simulation and gives an overview of the physical phenomena that were considered in the simulations. These are the deposition of a stochastic powder bed, the description of beam absorption, heat conduction, melting of the powder layer, melt pool dynamics, and solidification. 

In order to capture powder bed-related statistical processes in the simulations, a built height of many layers is necessary, which leads to very long simulation times. To reduce the simulation time, a grid coarsening algorithm was introduced by Bauereiß [43], where hydrodynamic calculations are only done within the finest grid. If only heat conduction is present, the spatial and temporal resolution is decreased. The finest spatial and temporal resolution of the simulations are 5 μm and 200 ns.

The width of the simulation domain was 6.25 whereby 5 mm were used for melting. Because the melt pool size reaches a quasi steady state after a few scan lines, side as well as bulk melting effects are covered by the reduced simulation domain. One hundred layers were built with a layer thickness and line offset of 50 μm as in experiments. The Gaussian-shaped beam moves from a virtual third dimension perpendicular through the simulation plane in a snake-like hatching strategy starting from right to left. The beam moves 15 mm per scan line and it crosses the simulation plane 101 times per layer. The simulation plane is placed in the middle of the scan lines. The 2D representation of the thermal field is more accurate when the electron beam moves perpendicular through the simulation plane. The heat conduction in the beam direction is less important since the beam movement is faster than the diffusive conduction. If the electron beam would move parallel through the simulation plane, the conduction out of the plane would be omitted and the temperature overestimated. Therefore, the beam movement is kept perpendicular to the simulation plane by rotating the hatching direction about 180${}^{\circ }$ each layer. Beam parameters were chosen in the same range as in experiments. However, velocities lower than 2 m/s were not used for the process map, because of the longer simulation times associated with small velocities.

As in the experiments, the parts were built on support structures. Therefore, the area density of the cylindrical supports beneath the samples in experiments was translated to a line density for the 2D simulations. This was done to obtain an average heat flux through the supports that is comparable with experiments. The width and the quantity of supports were adjusted, while the support density was preserved. Three supports with a width of 50 μm were placed beneath the built area for every simulation.

The process related effect of beam widening with increasing beam power was considered according to empirical data. The material properties used for the simulations are given in Table 1. The thermal conductivity was approximated by a linear fit in the solid and liquid phase [44]. All other material parameters were constant. The temperature of the powder bed was assumed to be higher than the measured temperature below the build platform. Therefore, an initial temperature of 827 ${}^{\circ }C$ was used in simulations. At the beginning of each layer the whole domain was reset to this temperature.

The random powder bed in the simulations was generated with the rain drop algorithm [50]. A powder particle size distribution based on the experimental distribution was used. Each particle falls downward at a random position until it makes contact with another particle or solidified material. Then the vertical position is again minimized by rolling around its circumference. If the center of the powder particle exceeds the current layer, the particle is rejected. When the powder layer is filled, random particles are removed until the defined powder bulk density is reached. The powder bulk density $\rho_{\mathrm{pow}}$ of the simulations for validation was assumed to be 55% in accordance with experimental data. For the numerical experiments, it was varied between 40% and 60% to investigate its influence on the final part density.

The relative density in the simulations was determined in an area of 4 × 4.6 mm${}^{2}$; i.e., with a horizontal distance of 500 μm from the side surfaces of the built samples and a vertical distance of 200 μm from the initial minimum and final maximum powder height. For statistical analysis, the area was divided in smaller sections along the vertical direction with a height of 100 μm. Finally, the average and standard deviation of the relative density for all sections were evaluated.

## 3. Results and Discussion

### 3.1. Temporal Evolution of Multi-Layer Manufacturing

**Figure 2:**
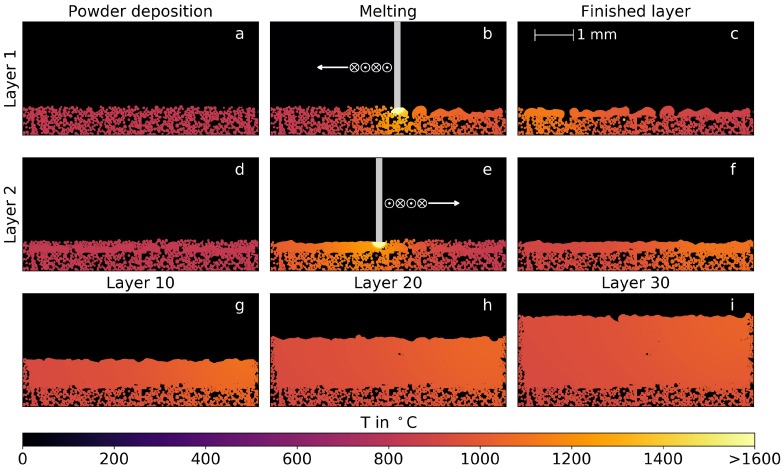
Temporal evolution of a simulated multi-layer building process. The deposited powder, the melting step, and the finished layer are shown for the first (**a**–**c**) and the second layer (**d**–**f**). Additionally, intermediate building heights after 10, 20, and 30 layers are illustrated (**h**–**i**).

The layer-by-layer buildup of samples is illustrated in Figure 2, where the temperature field is depicted at different times during the simulation.

The simulation applies a line energy of 120 J/m, a scan velocity of 1 m/s, and a powder bulk density of 55%. The first (a–c) and second (d–f) layers are shown. The initial powder bed with a building temperature of 827 ${}^{\circ }C$ (a) is melted by the electron beam that crosses the simulation plane vertically with a lateral movement from right to left (b). The melted material consolidates directly on the powder bed. Surface tension and wetting separate the melted areas and an irregular melt line remains. The residual heat is still clearly visible when the layer is finished (c). The new powder layer is applied up to the nominal powder height and the whole domain is reset to the building temperature (d). The second layer is melted with a lateral movement from left to right. With sufficient energy, a part of the previous layer is remelted, leading to a much more regular melt pool shape in comparison with the first layer (e) and finally to a more even surface (f). This procedure is repeated for a desired number of layers as illustrated for several intermediate building heights (g–i).

### 3.2. Comparison between Experiment and Simulation

**Figure 3:**
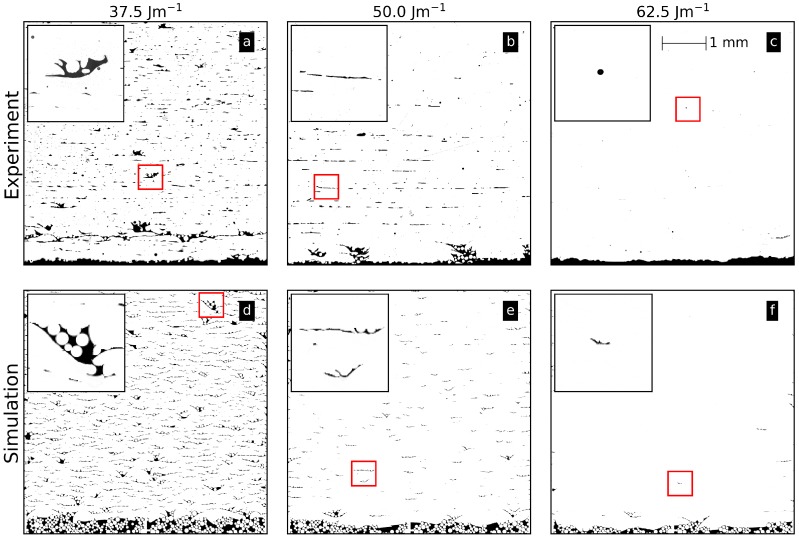
Comparison between simulation and experimental microsections for different line energies and a constant scan velocity of 20 m/s. The insets depict an enlarged area of multi-layer binding faults (**a**,**d**), single-layer binding faults (**b**,**e**,**f**), and gas pores (**c**).

Figure 3 shows three microsections from experimental Ti-6Al-4V samples and corresponding numerical results for a constant scan velocity of 20 m/s and different line energies. The parameters demonstrate the transition from porous to dense parts. The illustrated area of 5 × 5 mm${}^{2}$ is necessary to obtain an impression of the statistical occurrence of different defect features.

Generally, three kinds of defects are apparent in Figure 3: single layer binding faults (inset in b, e, and f), multi-layer binding faults (inset in a and d), and gas pores (inset in c). For the lowest line energy (a and d), many single layer binding faults occur. There is no sound connection between the layers established because the energy input is too low to remelt the previous layer. Additionally, multi-layer binding faults occur over a few layers. Their formation will be discussed in Section 3.4. Due to a process-related instability during manufacturing at approximately 1 mm height, a higher porosity compared to the rest of the sample is visible (a). In the numerical results, the multi-layer binding faults are still filled with residual powder particles (inset in d) in contrast to the experimental samples, where the particles drop out during preparation. Overall, the statistical occurrence of both defects is similar for the experimental and numerical results. For a line energy of 50 J/m (b and e), the multi-layer binding faults have almost vanished due to the higher energy input leading to deeper and larger melt pools. For the highest line energy (c), only some spherical pores due to residual gas in the powder particles and rare single layer binding faults can be detected in experiments. In the simulation, gas pores cannot be reproduced, because gas inside the powder particles is not considered. However, occasionally short single layer binding faults can also be found (inset in f).

In experiments, the bottom of the sample exhibits an increased porosity for a line energy of 50 J/m (b). Because the samples are built on support structures, the beam melts directly into the powder bed between the supports in the first layers. Thus, the bottom surface becomes more irregular due to an uneven powder bed surface. This effect can also be seen in all the numerical samples. Altogether, the simulations capture the main features from experiments.

### 3.3. Experimental and Numerical Process Window

**Figure 4:**
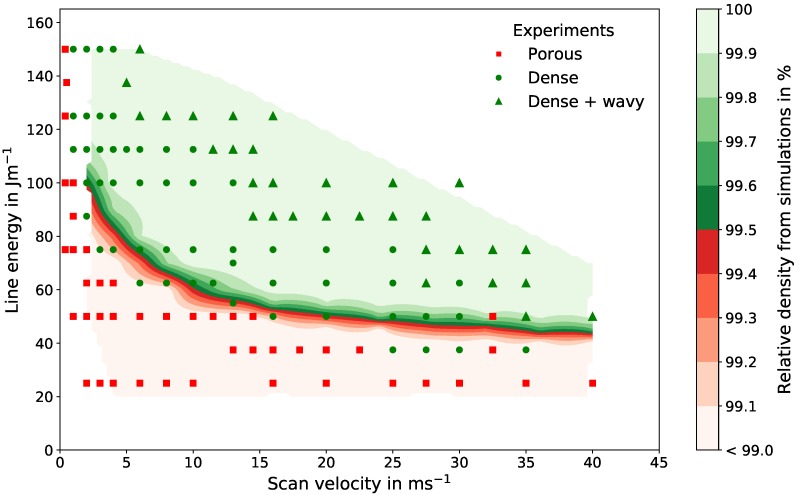
Process window for selective electron beam melted Ti-6Al-4V with a line offset and nominal powder layer thickness of 50 μm and a powder bulk density of 55%. Experimental results are classified into porous (red squares), dense (green circles), and wavy (green triangles). Numerical results are represented by a relative density map.

Figure 4 shows the experimental process window (symbols) and the relative density map obtained from simulations for a powder bulk density of 55%. The lower limit in the process window representing the transition from porous to dense parts is defined by a minimum relative density of 99.5%. The minimum required line energy for dense parts decreases with increasing scan velocity due to shorter return times of the beam resulting in less thermal losses [4]. This effect becomes less important for high scan velocities, where a minimum line energy of approximately 50 J/m for velocities above 15 m/s is reached.

The transition from porous to dense parts in the experiments fits well with the numerical results. For higher scan velocities above 25 m/s, the numerical prediction slightly overestimates the porosity border. However, for these velocities the experimental results are spread: between parameters for dense parts, some porous and wavy samples occur, which indicate process-related instabilities. For small velocities below 10 m/s, the simulations predict a transition at slightly higher line energies.

The upper limit in the process window is determined by the surface texture due to evaporation effects. The recoil pressure during evaporation leads to an increased melt pool movement and finally to a rough and uneven surface on the top of the samples [38], which should be avoided. Since evaporation effects are excluded in the simulations for this paper, the upper limit will not be further discussed.

Summarizing, the results from Figure 3 and Figure 4 demonstrate the predictive power of the numerical framework, since the model only relies on physical parameters, such as material properties, and no fitting parameters. The porosity features and statistics as well as the lower limit of the process window are well reproduced by the software.

### 3.4. Influence of the Powder Bulk Density on Defect Evolution

In this section, the sensitivity of the process window for varying powder bulk densities is investigated. As seen in Figure 4, the minimum line energy required for dense parts varies around 50 J/m for high scan velocities. Therefore, a constant scan velocity of 24 m/s and line energies between 40 J/m and 60 J/m are chosen.

**Figure 5:**
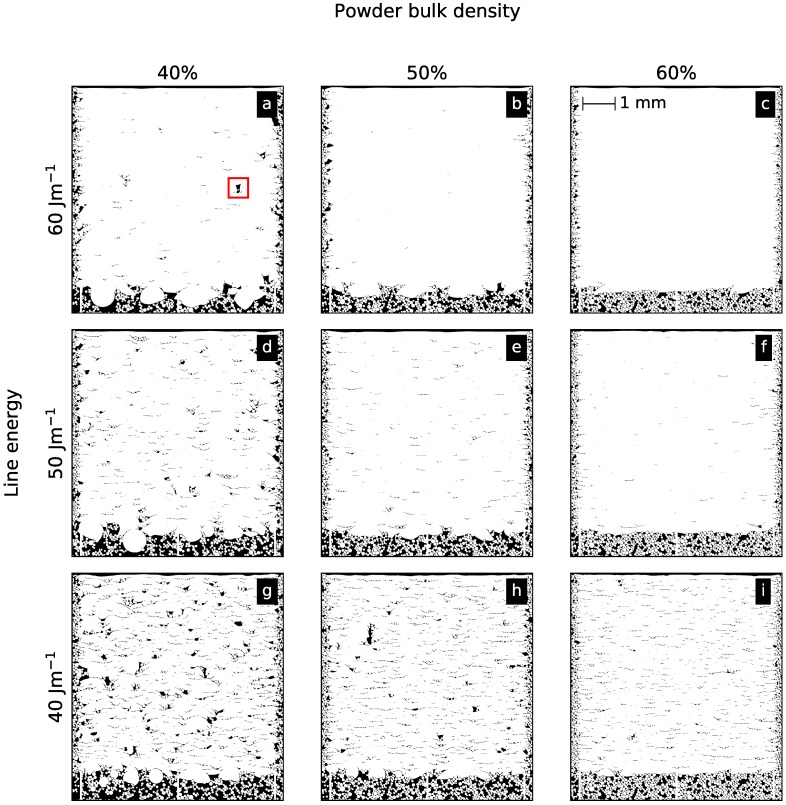
Numerical results for Ti-6Al-4V for powder bulk densities between 40% and 60% for a constant scan velocity of 24 m/s and different line energies of 40 (**g**,**h**,**i**), 50 (**d**,**e**,**f**) and 60 J/m (**a**,**b**,**c**). A multi-layer binding fault in (**a**) is highlighted for further discussion.

Figure 5 illustrates numerical results for powder bulk densities of 40%, 50% and 60%. For the lowest line energy of 40 J/m and powder bulk density of 40%, many multi-layer binding faults are present (g). With increasing the powder bulk density to 50% (h), multi-layer binding faults occur less often. Nevertheless, due to the stochastic powder bed, a channel-like defect ranging over 10 layers appears in the upper half of the domain. Note that a build height of several millimeters is necessary to capture these rarely occurring events. The amount of multi-layer binding faults further decreases for 60% powder bulk density (i). For a powder bulk density of 40%, increasing the line energy reduces the number of defects (a,d). With increasing line energy and powder bulk density, single and multi-layer binding faults occur more rarely until the parts for a powder bulk density of 50% and 60% show no multi-layer binding faults (b–f). For the highest line energy and the highest bulk density, the sample is completely dense without any defect (c).

One reason for a higher probability of binding faults with lower powder bulk densities is an increased effective layer thickness $l_{\mathrm{eff}}$. It can be defined with the nominal layer thickness $l_{\mathrm{nom}}$ and the powder bulk density $\rho_{\mathrm{pow}}$ as $l_{\mathrm{eff}}=l_{\mathrm{nom}}/\rho_{\mathrm{pow}}$. Therefore, the effective layer thickness for a powder bulk density of 40% is about 2.5 times higher than the nominal layer thickness, whereas for a powder bulk density of 60% the effective layer thickness is only increased by a factor of 1.7. Although the amount of material deposited in one layer is approximately the same, the local powder bulk density varies. Thus, at positions with a locally dense powder arrangement and a high effective layer thickness, the melt pool may not range deep enough to prevent binding faults. Consequently, lower powder bulk densities increase the stochastic variations in the powder bed and are therefore more prone to defects.

**Figure 6:**
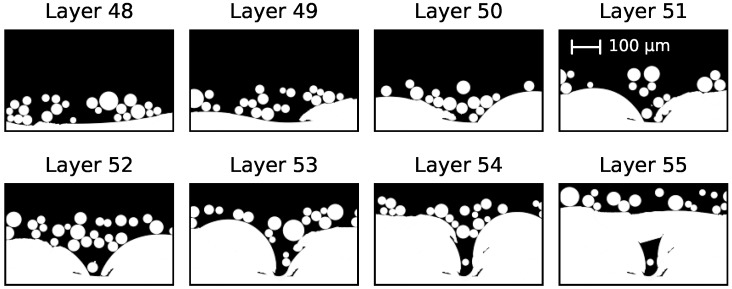
Evolution of a multi-layer binding fault through several layers. A detailed section of Figure 5a with a scan velocity of 24 m/s, a line energy of 60 J/m, and a powder bulk density of 40% is shown.

Another reason for a higher probability of defects is the movement of the melt on an uneven powder bed surface. The main driving force determining melt pool dynamics are capillary forces and wetting caused by the surface tension of the melt. If the powder bulk density decreases, the mean distance between the particles increases (i.e., the probability for gaps between the powder particles in a layer rises). This results in balling and the formation of extrusions and intrusions, because the melt is pulled away from regions where no wettable surfaces are present. A pronounced horizontal movement causes a much more irregular surface of the melted powder layer [3]. This can lead to cavities, which is demonstrated in Figure 6 for a small section of Figure 5a with a scan velocity of 24 m/s, a line energy of 60 J/m, and a powder bulk density of 40%. After melting layer 48, two adjacent convex surfaces are initialized. Due to the loose powder, the cavity between them is not closed until layer 54 is melted. Here, the powder particles lie densely enough on top of the cavity to close it. In the worst case such gaps lead to channel-like porosities that propagate through tens of layers (see Figure 5h) [34].

Lee and Zhang [33] demonstrated with numerical studies a pronounced balling within a single melt track due to a lack of particles in the powder layer. They concluded that it was possible to avoid balling effects by increasing the powder bulk density. A similar increased irregularity in the solidified material of single melt tracks is reported by Körner et al. [15]. Qiu et al. [51] stated that a stable melt flow is beneficial for the surface roughness and finally for the relative density. All these findings are in accordance with the present results. The unpredictability in the powder bed decreases the process reliability and reproducibility. Therefore, the required beam powers must be raised to establish melt pool sizes larger than the scale of the powder bed irregularities. Consequently, it was suggested to have a powder bulk density in every layer which was as high as possible [15,25,33,52]. For higher bulk densities, the surface becomes more sound, and accordingly the melt pool shape is smooth [15], leading to a more stable and predictable process.

Another effect that is visible in Figure 5—especially for a powder bulk density of 40%—is an extremely uneven surface at the bottom of the melted area. Many spherical features evolve due to balling. This effect decreases dramatically for higher powder bulk densities. Equally, the side surfaces of the parts are more regular for higher powder densities. In order to reduce surface roughness, special scan strategies near the contour of the build sample are used (e.g., by decreasing the energy input in the first layer, the mean melt depth in the powder is decreased. However, these strategies do not prevent balling and therefore irregularities of the surfaces remain.

### 3.5. Influence of the Powder Bulk Density on the Process Window

**Figure 7:**
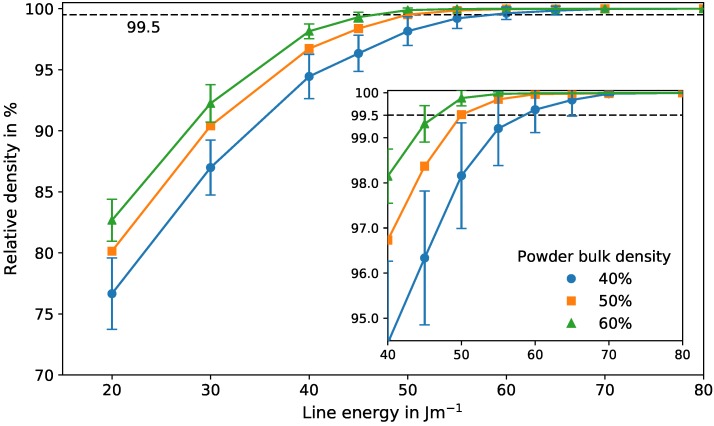
Relative density for different powder bulk densities for a constant scan velocity of 24 m/s and different line energies between 20 and 80 J/m with standard deviations. For convenience, the standard deviation for the results with a powder bulk density of 50% is not shown. The inset shows the regime for a relative density between 95% and 100%. The lines serve only as a guide to the eye.

The impact of the powder bulk density on the relative density of the final parts is demonstrated in Figure 7. It shows the relative density for different line energies with varying powder bulk densities for a constant scan velocity of 24 m/s. With increasing powder bulk density, the relative density increases accordingly for constant line energies. Additionally, the standard deviation decreases for higher bulk densities, reflecting less uncertainty during the process due to unfavored powder arrangements. Generally, low deviations are desired for high reliability and reproducibility. Regardless of the powder bulk density, the deviation also decreases with increasing line energies. Due to larger melt pools for higher line energies, irregularities in the powder layer can be compensated better, resulting in less porosity.

The powder bulk density has a strong influence on the transition region from porous to dense. The minimum line energy necessary to produce dense parts with a minimum relative density of 99.5% increases with decreasing powder bulk density. Comparing the results for a powder bulk density of 60% and 40%, the required energy increases by approximately 26%. This is true for the mean relative density. For reliable parameters that guarantee dense parts, a conservative estimation needs to be made by taking the standard deviation into account. Therefore, the required energy for dense parts increases even by 33% due to the higher deviations for lower powder densities.

Assuming that these effects are similar for different scan velocities, one can conclude: Since an increasing powder bulk density decreases the minimum line energy needed to build dense parts, the lower limit of the process window decreases with increasing powder densities. The upper limit of the process window, however, is determined mainly by evaporation effects and remains constant. Therefore, a higher powder bulk density leads to broadening of the parameter range for dense parts. In Figure 4, it can be seen that the line energy range between the lower and upper limit of the process window decreases with increasing scan velocities until the window is closed. Thus, the process window closes later if its lower limit descends. Consequently, dense parts can still be produced at higher scan velocities with higher powder bulk densities. This can shorten production times.

A possible strategy to improve the powder bulk density is to adjust the powder shape and size distribution. For example, a bimodal powder mixture can be beneficial for increasing the powder density [53]. Additionally, the flowability of the powder should be high to provide a homogeneous powder layer after deposition. Another way is to alter the deposition method. One possibility that is investigated is the usage of a vibrating powder roller to improve the powder arrangement [54].

## 4. Conclusions

In the present work, the predictive capability of our numerical framework for powder bed fusion additive manufacturing processes was examined. Simulations with the successive building of 100 layers with 101 scan lines each were performed to capture the porosity statistics and surface quality found in experiments due to the stochastic powder bed. Qualitatively, the simulations showed a good agreement with the porosity features and their statistical occurrence found in experiments for selective electron beam melted Ti-6Al-4V. Additionally, the surface structure could be reproduced. A numerically determined relative density map predicted the transition from porous to dense parts (minimum relative density of 99.5%) in the corresponding experimental process window sufficiently well.

Furthermore, the sensitivity of the relative density of built parts for varying powder bulk densities was demonstrated. The results show that the powder bulk density has a strong influence on the surface quality and the energy required to produce dense parts. For lower powder bulk densities the surface roughness increases remarkably. Moreover, by decreasing the powder bulk density from 60% to 40%, about 33% more energy is needed to produce dense parts. This is due to a higher probability of an irregular melt pool shape with decreased powder densities. Locally, an irregular powder arrangement may trigger the evolution of multi-layer binding faults ranging over several layers as a consequence of an unstable melt pool. This also leads to higher deviations in part densities, which further increases the energy input required for reliable dense parts. Thus, energy costs can be reduced with higher powder bulk densities.

Finally, lower powder bulk densities shrink the process window by shifting the lower boundary towards higher beam powers and by increasing the uncertainty of this boundary. Therefore, the process stability, reliability, and reproducibility can be increased by using an optimal powder with a powder bulk density as high as possible. This is crucial for lowering the amount of rejects during production.

## Figures and Tables

**Table 1:** Material properties of Ti-6Al-4V used in simulations.

Parameter	Value	Reference
Density	4122 $kg/m^{3}$	[45]
Solidus temperature	1878 K	[46]
Liquidus temperature	1928 K	[46]
Boiling temperature	3315 K	[47]
Dynamic viscosity	4.76 mPas	[48]
Surface tension	1.52 N/m	[48]
Specific heat (liquid)	670 J/K	[44]
Specific heat (solid)	1126 J/K	[44]
Thermal conductivity (solid) at 1200 K	17.2 W/m/K	[44]
Thermal conductivity (solid) at 1900 K	27.4 W/m/K	[44]
Thermal conductivity (liquid) at 2100 K	31.8 W/m/K	[44]
Thermal conductivity (liquid) at 2600 K	40.9 W/m/K	[44]
Latent heat of fusion	290 kJ/K	[44]
Latent heat of vaporization	9820 kJ/K	[49]
